# Assessment of Professional Oral Hygiene Care Needs Among Adolescents in Southeastern Serbia: A Clinical and Questionnaire-Based Study

**DOI:** 10.3390/healthcare14203389

**Published:** 2026-10-12

**Authors:** Ana Stojanović, Branislava Stojković, Zorana Veličković, Aleksandra Jovanović, Milica Kostić, Milica Petrović, Nikola M. Stojanović

**Affiliations:** 1 Department of Dentistry, Medical Faculty Niš, University of Niš, 18000 Niš, Serbia; branislava.stojkovic@medfak.ni.ac.rs (B.S.); zorana.velickovic@medfak.ni.ac.rs (Z.V.); aleksandra.jovanović@medfak.ni.ac.rs (A.J.); milicakosticcc3@gmail.com (M.K.); milica.petrovic@medfak.ni.ac.rs (M.P.); 2 Center for Mental Health Protection, University Clinical Centre Niš, 18000 Niš, Serbia; nikola.st90@yahoo.com

**Keywords:** adolescents, gingivitis, oral hygiene, professional oral hygiene

## Abstract

**Background/Objectives**: Adolescence is an important period for the development and consolidation of oral hygiene habits that may continue to influence oral health-related behaviors into adulthood. Inadequate oral hygiene may contribute to impaired gingival health and increased need for professional oral hygiene care. The aim of this study was to assess the need for professional oral hygiene care among adolescents in southeastern Serbia by evaluating their oral hygiene practices, oral hygiene status, and gingival health. **Methods**: This cross-sectional study included adolescents aged 14–18 years from southeastern Serbia. Data were collected using a questionnaire assessing oral hygiene practices, combined with a clinical examination of oral hygiene status using the Simplified Oral Hygiene Index (OHI-S) and gingival health using the Gingival Index (GI). The statistical analysis included descriptive statistics, Spearman’s correlation analysis, and linear regression analysis. **Results**: A total of 150 patients met the criteria for this study. The mean OHI-S and GI scores were 1.57 ± 0.87 and 1.80 ± 0.96, respectively. A total of 28.67% of participants reported gum bleeding during toothbrushing. During the 12 months preceding the study, 82% of respondents had received professional oral hygiene care only once. A statistically significant correlation was found between the GI and OHI (r_s_ = 0.769, *p* < 0.001). OHI values were significantly inversely correlated with both the frequency and duration of oral hygiene (r_s_ = −0.188, *p* = 0.022 and r_s_ = −0.186, *p* = 0.023, respectively). Univariate regression analysis identified oral hygiene frequency and gum bleeding as the most significant factor associated with gingivitis. The multivariate model showed that the GI was significantly associated with gender (*p* = 0.025), brushing twice daily (*p* = 0.038), gum bleeding (*p* = 0.003), and brushing technique (*p* = 0.037). **Conclusions:** Relatively high GI values were observed and significantly correlated with OHI-S scores, while toothbrushing frequency, gum bleeding, gender, and brushing technique were associated with the GI. These findings suggest a potential role for professional oral hygiene care and oral health education in supporting gingival health among adolescents in southeastern Serbia.

## 1. Introduction

Adolescence is a specific developmental period preceded by childhood, which represents the transition from childhood to adulthood. It lasts approximately from 12 to 18 years of age and is accompanied by numerous physical, cognitive, emotional, and social changes [1]. These changes are generally universal for all adolescents but can manifest in different ways. During this period, psychological maturation is accompanied by numerous body changes as well as changes in behavior. It is not uncommon for unhealthy habits to emerge at this time, which can negatively affect the general, including oral, health of adolescents and which can grow into potential problems later in life. This largely applies to oral hygiene habits, which can negatively affect the health of the gingiva and the supporting tissues of the teeth, as well as overall oral health. Oral health during adolescence may be influenced by a range of factors, including oral hygiene and health-related behaviors, hormonal changes, dietary habits, socio-economic factors, and selected general health-related conditions. Therefore, adolescence may be regarded as an important period for the development and consolidation of oral hygiene behaviors, which may contribute to the maintenance of gingival health over time [2].

Epidemiological studies show a high prevalence of gingival disease among adolescents worldwide, which ranges from 5 to 95% depending on age and the studied population [3,4]. The high prevalence of this oral disease, usually in less developed countries, and the negative impact of the disease on the quality of life of adolescents undoubtedly confirm its great public health and socio-economic significance [5]. This variability may be partly attributed to differences in the populations examined, as well as to substantial methodological heterogeneity, particularly regarding the diagnostic criteria, indices, and thresholds used to define gingivitis [6]. Unfortunately, data on the prevalence of this disease for Serbia, including southeastern Serbia, are scarce.

Etiological factors contributing to the high prevalence of gingivitis among adolescents are numerous [7,8]. However, gingivitis is primarily a biofilm-induced disease which is why oral hygiene plays an essential role in its etiology. In adolescents, hormonal changes are also of particular importance, as increased levels of sex steroid hormones during puberty may modify the inflammatory response of gingival tissues to dental biofilm, potentially contributing to increased gingival inflammation [9]. Gingival inflammation, particularly when accompanied by edema and tissue enlargement, may favor dental biofilm retention and accumulation, which can contribute to the persistence and exacerbation of hyperemia and inflammatory changes in the gingival tissues [10]. Data from the available literature on the awareness of adolescents and their habits regarding oral hygiene show significant variability [8,11,12]. In order to explain the observed differences among the studied groups, a large number of factors were taken into account [13,14,15]. The largest number of factors are related to the oral hygiene habits of adolescents and their oral hygiene status. It is well documented that the frequency, duration, and technique of brushing teeth and the supporting apparatus are directly related to the oral hygiene status of adolescents [8,16,17,18]. However, in maintaining oral hygiene, adolescents mainly rely on their own, previously adopted habits. Nevertheless, adequate home oral hygiene combined with regular professional oral hygiene care represents an important component in the maintenance of gingival health, alongside other biological, behavioral, and socio-economic factors that may influence gingival health during adolescence [19]. Regular dental check-ups provide an opportunity to assess oral and gingival health and, when indicated, to perform professional oral hygiene procedures, including professional removal of dental biofilm. These procedures should be distinguished from daily home oral hygiene practices and from oral hygiene instruction and motivation, which aim to support the development and maintenance of adequate self-care habits. Once gingival inflammation has developed in response to dental biofilm, edematous and hyperemic gingiva may make it more difficult to maintain adequate oral hygiene, potentially contributing to further biofilm accumulation. Therefore, professional oral hygiene, which also includes patient education on proper oral hygiene maintenance techniques, thereby improving acquired daily habits, plays a key role in ensuring long-term preservation of oral health. Professional oral hygiene refers to a set of professionally performed preventive measures aimed at controlling dental biofilm and calculus and supporting effective personal oral hygiene. These measures include the professional mechanical removal of dental biofilm and calculus, polishing of tooth surfaces, oral hygiene instruction, and patient motivation to improve and maintain effective daily oral hygiene practices. Professional oral hygiene is intended to complement, rather than replace, patients’ daily self-performed oral hygiene [20]. The need for professional oral hygiene should be determined based on a comprehensive assessment of oral hygiene habits, gingival and periodontal status, and relevant clinical and behavioral factors [21]. Previous studies suggest that professional oral hygiene should be performed at approximately six-month intervals in individuals with healthy gingiva, whereas more frequent visits, typically every three to four months, may be recommended for individuals with gingivitis [22,23]. Regular preventive dental visits at intervals of three to six months may be appropriate for some adolescents; however, the frequency of recall and professional oral hygiene care should be individualized according to the patient’s clinical status, risk profile, and individual needs [24].

Poor oral hygiene and the high prevalence of gingival inflammation among adolescents remain important concerns in dental practice. Despite the important role of professional oral hygiene care in maintaining gingival health, data on the use and potential need for such care among adolescents remain limited, particularly in Serbia and the southeastern region of the country. This lack of population-specific evidence may limit the development of targeted preventive strategies and individualized oral hygiene care recommendations. Further research is therefore needed to characterize oral hygiene practices, clinical oral hygiene status, and gingival health in this population and to provide evidence to support preventive care.

Based on our clinical experience with the gingival health of adolescents in southeastern Serbia, we hypothesized that there is a need for professional oral hygiene care in this population. Accordingly, the aim of this study was to assess the need for professional oral hygiene among adolescents in southeastern Serbia based on their oral hygiene practices, oral hygiene status, and gingival health.

## 2. Materials and Methods

### 2.1. Ethics Statement

This cross-sectional study was conducted in accordance with all principles of the Declaration of Helsinki and was approved by the Ethics Committee of the Faculty of Medicine, University of Niš (decision number 12-4370-1/2-5). It included patients from the Department of Preventive and Pediatric Dentistry, Clinic of Dental Medicine, Faculty of Medicine, University of Niš, Serbia. The director of the institution where the research was conducted, as well as the head of the Service for Preventive and Pediatric Dentistry, were informed orally and in writing about the procedure and goal of the research, the method of data collection, and the protection of the respondents’ identity, after which they gave written consent for participation in the research. Also, before the start of the research, all respondents, or their companions (parents or guardians), were given an information sheet with a description of the research, to which they gave their consent for voluntary participation in the research with their signature and agreement. During the research, all security measures regarding the protection of the rights and personal data of the respondents participating in the research were taken. The identity of all respondents was marked numerically, thus preserving their privacy and anonymity.

### 2.2. Study Design and Participants

To realize our goal, a cross-sectional study was conducted from August 2025 to February 2026.

### 2.3. Inclusion and Exclusion Criteria

Inclusion criteria were as follows: (1) healthy patients aged 14–18 years; (2) patients with a Community Periodontal Index of Treatment Needs (CPITN) value of 0, 1, or 2; (3) patients with no periodontal pockets (score 0); (4) patients without systemic and acute infectious diseases in their personal history; patients not in receipt of antibiotic or chronic therapy; patients who did not consume supplements (vitamins, etc.); (5) patients who completely and adequately filled out the survey form; and (6) patients who agreed to participate in the study.

Exclusion criteria were as follows: (1) patients undergoing orthodontic therapy; (2) smokers; (3) reported alcohol use; (4) patients with fibromatous and proliferative gingivitis. All respondents were allowed to withdraw from participation in the research at their own request at any time, without any consequences.

Selection of respondents was performed based on medical/dental records, clinical examination of the respondents, and data obtained through patient surveys.

### 2.4. Data Collection

Research data were collected through participant surveys and clinical examination.

### 2.5. Surveying Participants

To collect data, a modification of the survey by Sbricoli et al. (2022) was used [18]. The survey form consisted of five groups of questions. The first group of questions included basic data about the respondents (date of birth, gender, school attended, data on general health status and medication use, orthodontic therapy, cigarette consumption, etc.). The second group included general questions related to their first visit to the dentist. The third group included questions on oral hygiene habits at home, while the fourth group included questions on the respondent’s awareness and education regarding professional oral hygiene. The fifth group of questions referred to instructions regarding oral hygiene maintenance and the respondent’s attitude on that issue. All questions in the survey were of the closed type, with predefined answers. The possible presence of parents was not expected to influence participants’ responses, as the questionnaires were administered individually and completed only in the presence of the examining dentist, without parental involvement.

### 2.6. Clinical Examination of Participants

After the survey, each participant underwent a clinical examination where the condition of the tooth-supporting tissue, gingival health status, and oral hygiene status of the respondent was recorded. All clinical examinations were performed by a trained examiner who was a specialist in Preventive and Pediatric Dentistry with extensive experience in conducting epidemiological studies. Clinical dental examinations were performed with participants seated in a dental chair under artificial lighting, using a dental mirror, a dental probe, and a calibrated periodontal probe (PQWBR—Hu Friedy Mfg. Inc., Chicago, IL, USA). The condition of the supporting apparatus was determined by the CPITN [25]. Gingival health status was assessed using the Mühlemann bleeding index [26], while oral hygiene status was assessed using the OHI-S [27]. Periodontal pocket depth was assessed by probing the periodontal sulcus/pocket using a calibrated periodontal probe. The probing depth was measured in millimeters from the gingival margin to the bottom of the periodontal pocket at the examined sites. For the assessment of the OHI-S, six surfaces of representative permanent teeth were examined: the buccal surfaces of the maxillary right and left first molars, the lingual surfaces of the mandibular right and left first molars, the labial surface of the maxillary right central incisor, and the labial surface of the mandibular left central incisor. The examination was performed using a dental probe and included only fully erupted teeth. The mean index value was calculated by summing the scores recorded for all examined representative teeth and dividing the total by the number of teeth examined. If a representative tooth was missing, it was not replaced by an adjacent tooth. The Mühlemann bleeding index was assessed using a blunt periodontal probe. For each tooth, the mesial and distal aspects of the interdental papilla were examined. Gingival bleeding was evaluated using a 5-point scoring system ranging from 0 to 4. The index value was calculated by summing the scores recorded for all examined teeth and dividing the total score by the number of teeth examined. The Community Periodontal Index of Treatment Needs (CPITN) was assessed using a blunt periodontal probe. Six representative teeth (16, 11, 26, 36, 31, and 46) were examined, as recommended for children and adolescents aged 7–19 years. Four sites around each tooth—mesial, distal, buccal, and lingual—were probed, and the highest CPITN score recorded for each tooth was used for the assessment. The mean CPITN value was calculated by summing the scores obtained for all examined representative teeth and dividing the total by the number of teeth examined. If a representative tooth was missing, it was not replaced by an adjacent tooth.

### 2.7. Sample Size

The sample size was calculated using G*Power version 3.1.9.7 for a one-tailed one-sample *t*-test (means: difference from constant [one-sample case]), with a significance level of 5% and a statistical power of 95%. The calculation was based on the bleeding index values reported in a previous study [28], in which the mean bleeding index was 0.64 ± 0.59, with a clinical threshold for an unfavorable outcome defined as ≥1. The minimum required sample size was calculated to be 31 participants. A total of 150 patients were included in the study to increase the precision of the estimates and to better account for variability in the assessed parameters.

### 2.8. Statistical Analysis

Statistical analyses of the collected data were performed using R software (version 4.5.1; R Foundation for Statistical Computing, Vienna, Austria) [29]. Within descriptive statistics, numerical data were presented by measures of central tendency (mean) and measures of variability (standard deviation). Within analytical statistics, methods were used to determine the significance of the obtained differences, as well as methods for testing the correlation of the tested variables. Comparison of categorical variables was performed using the χ^2^ test. Comparison of frequencies of the obtained data was also performed using the χ^2^ test. The mutual correlation of potential factors, as well as their correlation with the tested independent variables, were tested using the Spearman simple linear correlation coefficient. The potential associations between the Gingival Index (GI) and the investigated variables were assessed using univariable and multivariable linear regression analyses. All categorical variables were coded as binary variables, with one category designated as the reference category for each variable. Univariable linear regression analyses were initially performed to examine the associations between individual demographic and oral hygiene-related variables and the Gingival Index (GI). Variables considered clinically relevant to gingival health and the objectives of the study were subsequently included in the multivariable linear regression model. The null hypothesis was tested with a significance level of *p* < 0.05.

## 3. Results

**Figure 1:**
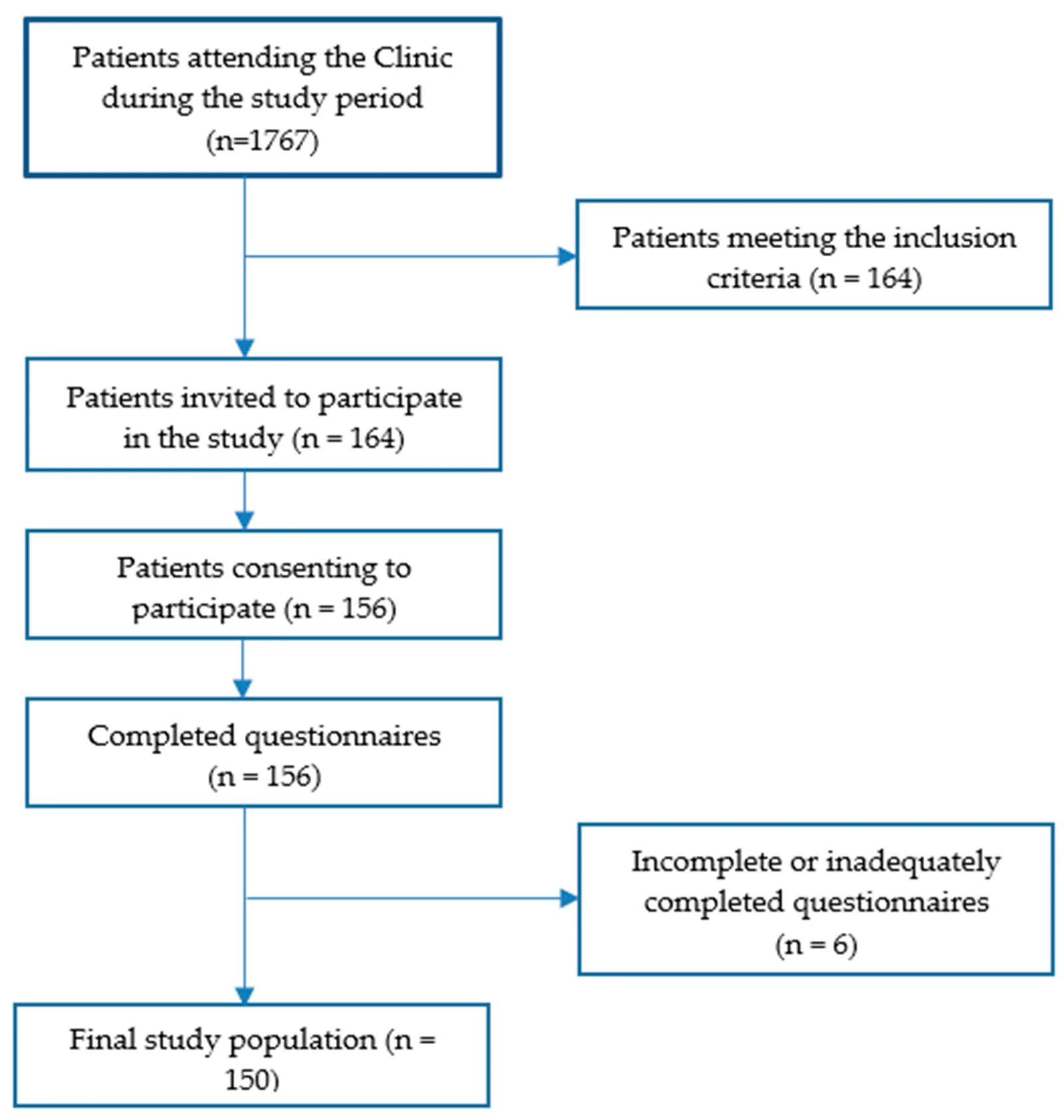
Flowchart of the study.

Out of 1767 patients, 150 participants met the inclusion criteria for the study (Figure 1).

The study included 64 boys and 86 girls, with an average age of 15.98 ± 1.29, with no significant difference in the gender of the participants (*p* = 0.07). A significantly higher number of patients included in the study were from grammar schools compared to other vocational schools (*p* < 0.001). The average OHI-S value of the participants was 1.57 ± 0.87, while the average GI value was 1.80 ± 0.96. Basic demographic and clinical characteristics of the participants are shown in Table 1.

A statistically significantly percentage of respondents had visited a dentist before, 92% of them (*p* < 0.001). Also, a statistically significant difference was recorded regarding the time of their first visit to the dentist. The highest percentage of respondents had visited the dentist for the first time between the ages of 5 and 9, 54.6% of them, and the least after the age of 10 (*p* < 0.001). Basic information about the participants’ first visit to the dentist is shown in Table 2.

All respondents pointed out that they had their own toothbrush. A statistically significant difference was recorded in the representation of toothbrush types (*p* < 0.001). A manual toothbrush was used by the highest percentage of respondents (83.3%; *p* < 0.001). Data on home oral hygiene maintenance in the study group are shown in Table 3. There was a statistically significant difference regarding the frequency of performing oral hygiene (*p* < 0.001), with the highest percentage of respondents brushing their teeth twice a day (morning and evening), 64.67% of them. The duration of tooth brushing for as many as 78.67% of respondents lasts from 1 to 3 min, and 60.67% of them brush their teeth with combined movements. Statistically significantly, respondents adopted their tooth brushing technique from their parents (41.33%) and dentists (40.67%), while the internet was the least represented source of information regarding tooth brushing technique (4.67%). Gum bleeding was reported by 28.67% of respondents. Besides a toothbrush, as many as 46.67% did not use any other means for maintaining oral hygiene. Interdental brushes and dental floss were the most used additional basic tools for maintaining oral hygiene (16% and 24%, respectively).

Sixty percent of respondents were advised to improve their oral hygiene. No statistically significant difference was registered regarding the respondents’ familiarity with the importance of professional oral hygiene (*p* = 0.07). During the previous 12 months, 82% of respondents had received professional oral hygiene care, but only once. As many as 92% of respondents pointed out that they wanted more information about the importance and possibilities of applying professional hygiene for oral disease prophylaxis. Data on professional oral hygiene in the study group are shown in Table 4.

It was determined that there is a statistically significant correlation between the GI and the OHI (r = 0.769, *p* < 0.001) (Table 5). Other clinical parameters were not statistically associated with the GI. The OHI values were significantly inversely correlated with the frequency and duration of oral hygiene (r = −0.188, *p* = 0.022 and r = −0.186, *p* = 0.023).

Univariable linear regression analysis showed that the frequency of tooth brushing and gum bleeding are the most significant factors associated with the GI (Table 6). In a multivariable model including age, gender, brushing teeth twice a day, brushing duration of 1 to 3 min, toothbrush type, gum bleeding, brushing technique, and dental visits in the last year, it was shown that the GI is statistically significantly associated with gender (*p* = 0.025), brushing teeth 2x daily (*p* = 0.038), gum bleeding (*p* = 0.003), and brushing technique (*p* = 0.037) (Table 7).

## 4. Discussion

Despite substantial progress in the prevention and management of gingivitis, this condition continues to pose a major public health and economic burden worldwide [30,31]. Gingivitis remains highly prevalent across different populations, with its prevalence increasing with age. Adolescence is considered a particularly vulnerable period for the development of gingival inflammation, as both clinical observations and epidemiological evidence have demonstrated increased gingival bleeding and inflammatory changes in this age group [8,11,32]. Gingivitis is a multifactorial condition in which dental biofilm represents the primary etiological factor, while its onset and clinical expression may be influenced by local oral conditions, hormonal changes, dietary and nutritional factors, systemic diseases, medications, tobacco and other substance use, socio-economic and behavioral factors, stress, genetic susceptibility, and individual host immune and inflammatory responses. Since gingivitis is primarily a biofilm-induced disease, its onset and progression are strongly associated with oral hygiene behaviors, making adolescence a critical period for the establishment of effective oral hygiene practices. Therefore, professional oral hygiene care during this vulnerable developmental period may play a crucial role in the primary prevention of gingival disease. Consequently, understanding the need for and utilization of professional oral hygiene care among adolescents is essential for the development of effective preventive oral health strategies. In light of these considerations, the aim of this study was to assess the need for professional oral hygiene among adolescents in southeastern Serbia based on their oral hygiene practices, oral hygiene status, and gingival health.

A clinical and questionnaire-based study was conducted. The study recorded relatively high GI values among the examined adolescents, consistent with findings reported in adolescent populations from different geographical regions [6,21]. Such findings confirm that this period is associated with a high risk of developing gingival inflammation. The GI was used in this study due to its simplicity, non-invasiveness, and high sensitivity in detecting early gingival inflammatory changes, as well as its value as an objective and clinically observable sign of inflammation. The obtained GI values may be related to several contributing factors such as dental visits, oral hygiene habits, etc. Most of the examined adolescents reported first visiting a dentist between the ages of 5 and 9, which is consistent with the findings of similar studies by Sbricoli et al. [18]. However, from a preventive dentistry perspective, this can be considered a relatively late age for a first dental visit. An early dental visit allows for timely education of the child and parents about the importance of oral health and age-appropriate preventive measures. The establishment of trust between the child and the dentist is a key determinant of positive oral health behaviors and long-term adherence to regular dental check-ups. Literature data from research conducted by Chapple et al. [21] and Alshahrani et al. [33] show that early preventive dental visits are directly linked to future gingivitis status. A small number of respondents in this study visited the dentist for the first time at a slightly older age, around puberty. Although these data do not necessarily indicate poorer oral hygiene, it can be assumed that for these respondents, oral hygiene habits were not adequately formed and that there is a need for additional education and professional guidance.

Relatively high OHI-S values were recorded among the examined adolescents, which is consistent with the findings reported by Vasconcelos et al. and James et al., but contrasts with the results of Reddy et al. and Tagelsir et al. [34,35,36,37]. Proper oral hygiene performance at home is of great importance for the prevention of caries and gingivoperiodontal diseases, where mechanical control of dental biofilm represents the basis of prevention. All respondents in this study owned a toothbrush, while the majority of participants stated that they brush their teeth twice a day and change their toothbrush every six months. The effectiveness of oral hygiene depends not only on the frequency of brushing but also on the correct technique and duration of brushing. Most respondents used a combination of horizontal, vertical, and circular motions when brushing their teeth. Almost half of the respondents did not use auxiliary aids for maintaining oral hygiene, while among those who did, dental floss was the most common. Most respondents also did not use mouthwash in their daily oral hygiene routine. The obtained results indicate different patterns of oral hygiene behavior among adolescents.

Furthermore, the study confirmed a significant correlation between the GI and OHI-S, while the OHI-S was significantly inversely correlated with both the frequency and duration of oral hygiene practices. Similar findings have been reported by other authors [38,39].

Univariable linear regression analysis showed that brushing teeth twice a day and gum bleeding were significantly associated with the GI. In the multivariable linear regression model including age, gender, brushing teeth twice a day, brushing duration of 1 to 3 min, toothbrush type, gum bleeding, brushing technique, and dental visits in the last year, the GI was significantly associated with gender, brushing teeth twice a day, gum bleeding, and brushing technique. When interpreting these results, the regression analysis findings should be taken into account, as gender, brushing frequency, and brushing technique were significantly associated with the GI and may contribute to the prediction of gingivitis, with females showing a higher probability of developing gingivitis. The higher prevalence of gingival inflammation observed among female adolescents may be attributed to hormonal changes during puberty, which can enhance the inflammatory response of gingival tissues to dental biofilm, as reported by Doufexi and Nichols, Kinane et al., and Elgasmi et al. [7,8,30]. The association between the GI and toothbrushing technique and frequency is of particular importance, as it clearly indicates a pronounced need for intensive and periodic professional oral hygiene interventions in order to prevent gingivitis. Questionnaire data showed that 82% of adolescents had received professional oral hygiene care only once during the preceding 12 months. As comparable data from the study region are currently unavailable, direct comparison with other populations within the region is not possible. Nevertheless, the observed low frequency of professional oral hygiene care suggests that such preventive services may be insufficiently utilized among adolescents in the study population.

The results of this study suggest that greater awareness of adequate oral hygiene practices and appropriate preventive dental care may be beneficial for maintaining gingival health and may indicate a potential need for regular professional oral hygiene care among adolescents in southeastern Serbia. Professional oral hygiene plays a significant role in removing dental biofilm and calculus from surfaces that are not accessible by daily oral hygiene measures, as well as in educating and motivating adolescents to properly maintain oral hygiene. Most respondents stated that they had received recommendations from their dentist for improving their oral hygiene, but more than half were not familiar with the term professional oral hygiene, which may partially explain the registered values of the OHI-S and the GI. Nevertheless, most respondents had received professional oral hygiene at least once during the previous year during a regular dental check-up, and a significant number of them showed interest in additional information about this procedure. The finding that 92% of participants expressed an interest in receiving additional information about professional oral hygiene suggests a substantial interest in further oral health education among the adolescents included in this study. Moreover, as parents and dentists were reported as the main sources of instruction on toothbrushing techniques, both may have an important role in supporting appropriate oral hygiene practices during adolescence. These findings highlight the relevance of oral health education and self-care guidance in this age group. The obtained results indicate the need for more intensive education of adolescents and a greater focus on preventive programs, since patient motivation, regular check-ups, and professional oral hygiene represent important measures in the prevention of gingivitis and the preservation of oral health.

Although inadequate oral hygiene is an important determinant of gingival inflammation, gingival health during adolescence may also be influenced by multiple factors, including pubertal hormonal changes, malocclusion, dietary and nutritional factors, lower socio-economic status, tobacco and alcohol use, vaping and other substance use, as well as acute and chronic systemic conditions; these factors were not assessed in the present study and should therefore be considered in future research [21].

Along with the sample size, one of the main limitations of the study relates to the fact that the data were obtained using a survey questionnaire based on respondents’ self-assessment. Certain data, especially those related to the early childhood period, may be based on respondents’ retrospective memory and may therefore be subject to bias and recall errors. In addition, a cross-sectional study was conducted, which represents an additional limitation, especially related to questions about the provision of professional oral hygiene. An additional limitation of the present study is that several factors that may influence gingival health and the development of gingivitis were not assessed, including dietary habits, socio-economic circumstances, environmental factors, and other relevant general health-related factors. In addition, dental caries was not assessed; therefore, the findings of the present study should not be generalized to the overall oral health status of the examined adolescents. Accordingly, the observed associations should be interpreted within the context of the variables assessed in the present study. In order to obtain more precise data, well-designed longitudinal studies are needed that include a larger number of respondents, using objective clinical indicators and data from medical records. In this way, the bias associated with self-assessment and the retrospective memory of the respondents would be reduced, thereby increasing the reliability of the results. The obtained data would significantly contribute to the improvement of preventive programs and the planning of measures for the prevention of gingivitis in children in the studied region.

## 5. Conclusions

The results of the present study support the relevance of preventive approaches aimed at maintaining adequate oral hygiene and gingival health among adolescents in the study population, and suggest the need to strengthen professional oral hygiene care. Oral health education and the promotion of effective self-care practices should be considered complementary components of preventive care for this population. Further studies involving larger and more representative samples are warranted to strengthen the evidence base and inform population-specific preventive strategies for adolescents in southeastern Serbia.

## Figures and Tables

**Table 1:** Basic demographic and clinical characteristics of the respondents.

Characteristics	N	Min–Max %; Mean ± SD	*p* ^1^
**Age**	150	14–18 years 15.98 ± 1.29	
**Gender**			
Male	64	42.6%	0.07
Female	86	57.4%	
**School**			
Medical vocational school	39	26%	*p* < 0.001
Gymnasium	79	53%	
Technical vocational school	32	21%	
**GI**	150	1.80 ± 0.96	
**OHI-S**	150	1.57 ± 0.87	

N—number; SD—standard deviation; *p*-value; ^1^ χ^2^ test.

**Table 2:** General data related to participants’ first visit to the dentist.

Characteristics	N	%	*p* ^1^
**Visited the dentist before**			
Yes	138	92%	*p* < 0.001
No	12	8%	
**First visit to the dentist**			
Before age of 4	52	34.4%	*p* < 0.001
Between 5 and 9 years	82	54.6%	
Between 10 and 12 years	8	5.5%	
After 13 years	8	5.5%	

N—number; *p*-value; ^1^ χ^2^ test.

**Table 3:** Data on home oral hygiene maintenance in the study group.

Characteristics	N	%	*p* ^1^
**Own toothbrush**			
Yes	150	100%	*p* < 0.001
No	0	0%	
**Type of toothbrush**			
Manual	125	83.3%	*p* < 0.001
Electric	24	16%	
Sonic	1	0.7%	
**Duration of toothbrush use**			
Up to 6 months	139	92.7%	*p* < 0.001
More than 6 months	11	7.3%	
**Frequency of oral hygiene**			
Less than once a day	2	1.33%	*p* < 0.001
Once a day (morning)	3	2%	
Once a day (evening)	10	6.67%	
Twice a day (morning and evening)	97	64.67%	
Three times a day	30	20%	
More than three times a day	8	5.33%	
**Duration of tooth brushing**			
At most a minute	7	4.67%	*p* < 0.001
From 1 to 3 min	118	78.67%	
Over 3 min	25	16.66%	
**Brushing motions**			
Horizontal (left–right)	11	7.33%	*p* < 0.001
Vertical (up–down)	29	19.33%	
Circular motions	19	12.67%	
Combination of all motions	91	60.67%	
**Tooth brushing technique informed by**			
Parent(s)	62	41.33%	*p* < 0.001
Independently	20	13.33%	
Dentist	61	40.67%	
Internet and social networks	7	4.67%	
**Gum bleeding during tooth brushing**			
Yes	43	28.67%	*p* < 0.001
No	107	71.33%	
**Additional tools**			
Interdental brushes	24	16%	*p* < 0.001
Dental floss	36	24%	
Toothpicks	19	12.67%	
Water pick	6	4%	
Tongue cleaner	23	15.33%	
None of the above	70	46.67%	
**Mouthwash**			
Yes	34	22.67	*p* < 0.001
No	116	77.33%	

N—number; *p*-value; ^1^ χ^2^ test.

**Table 4:** Data on professional oral hygiene in the study group.

Characteristics	N	%	*p* ^1^
**Advised to improve oral hygiene**			
Yes	90	60%	0.046
No	60	40%	
**Familiarity with the importance of professional oral hygiene**			
Yes	64	42.6%	0.07
No	86	57.4%	
**Receipt of professional oral hygiene in the last 12 months**			
Yes, only once	123	82%	*p* < 0.001
Yes, more than once	0	0%	
No	17	11.33%	
Do not remember	10	6.67%	
**Do they want to know more information about professional oral hygiene?**			
Yes	138	92%	*p* < 0.001
No	12	8%	

^1^ N—number.

**Table 5:** Spearman correlation analysis of the Gingival Index and oral hygiene-related variables.

		OHI-S	OH Frequency	OH Duration
GI	r_s_	0.769 **	−0.083	−0.098
	*p*	0.000	0.313	0.234
	n	150	150	150
OHI-S	r_s_	1	−0.188 *	−0.186 *
	*p*		0.022	0.023
	n		150	150
OH Frequency	r_s_		1	−0.017
	*p*			0.833
	n			150

r_s_—Spearman’s correlation coefficient; *p*-value; * *p* < 0.05; ** *p* < 0.001.

**Table 6:** Univariable linear regression analysis of home and professional oral hygiene variables associated with the Gingival Index (GI) using the enter method.

	Unstandardized Coefficients		Standardized Coefficients	95% CI		*p*
	B	SE	Beta			
(Constant)	2.345	0.282		1.787	2.903	0.000
Twice a day	−0.170	0.082	−0.168	−0.333	−0.008	0.040
(Constant)	1.448	0.356		0.744	2.152	0.000
1 to 3 min	0.188	0.194	0.079	−0.196	0.572	0.335
(Constant)	1.597	0.262		1.079	2.114	0.000
Type of toothbrush	0.160	0.214	0.062	−0.262	0.583	0.454
(Constant)	2.608	0.305		2.005	3.211	0.000
Gum bleeding	−0.481	0.172	−0.224	−0.821	−0.141	0.006
(Constant)	2.013	0.169		1.679	2.347	0.000
Combination of all motions	−0.082	0.054	−0.125	−0.189	0.024	0.128
(Constant)	1.763	0.187		1.393	2.133	0.000
Visit in the last year	0.017	0.135	0.010	−0.250	0.284	0.902

Dependent variable: GI. B = unstandardized regression coefficient; SE = standard error; Beta = standardized regression coefficient; 95% CI = 95% confidence interval; *p*-value.

**Table 7:** Multivariable linear regression analysis of demographic and oral hygiene-related variables associated with the Gingival Index (GI).

	Unstandardized Coefficients		Standardized Coefficients	95% Confidence Interval for B		*p*
	B	SE	Beta			
(Constant)	3.011	1.158		0.721	5.300	0.010
Age	0.033	0.063	0.045	−0.091	0.158	0.596
Gender	−0.362	0.159	−0.184	−0.677	−0.047	0.025
Brushing teeth twice a day	−0.170	0.081	−0.167	−0.330	−0.010	0.038
Brushing duration from 1 to 3 min	0.128	0.190	0.054	−0.247	0.504	0.500
Type of toothbrush	0.313	0.209	0.120	−0.099	0.726	0.135
Gum bleeding	−0.543	0.180	−0.253	−0.898	−0.188	0.003
Brushing technique	−0.113	0.053	−0.171	−0.218	−0.007	0.037
Visit in the last year	0.014	0.136	0.009	−0.254	0.282	0.917

Dependent variable: Gingival Index (GI). Predictors: age, gender, brushing teeth twice a day, brushing duration 1 to 3 min, type of toothbrush, gum bleeding, brushing technique, and visit in the last year. Adjusted R^2^ = 0.098. B = unstandardized regression coefficient; SE = standard error; Beta = standardized regression coefficient; 95% CI = 95% confidence interval; *p*-value.

## Data Availability

The data presented in this study are available on request from the corresponding author. The data analyzed in the present manuscript were obtained from minor participants and form part of a broader research project that is still ongoing, with additional analyses planned. Consequently, the original research database cannot be provided in its entirety, as it contains information and variables that extend beyond the scope of the present manuscript and are intended for further investigation. In addition, particular consideration must be given to protecting the confidentiality of data collected from minors.

## Abbreviations

The following abbreviations are used in this manuscript:

CPITN	Community Periodontal Index of Treatment Needs
OHI-S	The Simplified Oral Hygiene Index
GI	Gingival Index
